# Maximum occlusal bite force in pre-school children with different occlusal patterns

**DOI:** 10.4317/jced.55181

**Published:** 2018-11-01

**Authors:** Elham Abu-Alhaija, Arwa I. Owais, Hiba Obaid

**Affiliations:** 1 BDS, PhD, MOrth RCS (Ed.), FDS RCS(Ed.). Professor, Division of Orthodontics, Department of Preventive Dentistry, Faculty of Dentistry, Jordan University of Science and Technology, Irbid-Jordan; 2 BDS, MDent Sci, Dip(ABPed). Associate Professor, Division of Paediatric Dentistry, Department of Preventive Dentistry, Faculty of Dentistry, Jordan University of Science and Technology, Irbid-Jordan; 3 BDS, MDent Sci. Master student, Department of Preventive Dentistry, Faculty of Dentistry, Jordan University of Science and Technology, Irbid-Jordan

## Abstract

**Background:**

This study was carried out to record maximum occlusal bite force (MOBF) in pre-school children with different occlusal patterns.

**Material and Methods:**

A randomly selected sample of 1085 kindergarten children in primary dentition stage were selected. The age of subjects ranged between 3-6 years (averaged 4.90 ± 0.86 years). The subjects were divided into 3 groups according to molar relationship; flush terminal (n=335; 165 males and 170 females), distal step (n=450; 200 males and 250 females), mesial step (n=300; 150 males and 150 females) molar relationship. Clinical examination involved the record of molar relationship, overjet, overbite and the presence of wearing facets. Occlusal bite force was measured using a hydraulic occlusal force gauge.

**Results:**

The means of MOBF for the different occlusal relationship were: - 193.47N (± 60.98), 179.20N (±56.80) and 245.11N (±69.45) for flush terminal, mesial and distal step molar relationships, respectively. Significant differences between studied groups were detected (*P*<0.01; *P*<0.001). MOBF were higher in subjects with distal step molar relationship, increased overjet and increased overbite. Gender differences were detected in flush terminal and distal step molar relationships.

**Conclusions:**

MOBF was affected by the different occlusal relationships. Children with distal step and mesial step molar relationship had the highest and the lowest MOBF, respectively. MOBF was similar in children with/without wearing facets.

** Key words:**Occlusal, Bite force, primary, dentition, Pre-school.

## Introduction

Occlusal bite force (OBF) is one indicator of the functional state of the masticatory system that results from the action of jaw elevator muscles modified by the craniofacial biomechanics (1). Several factors have been suggested to affect OBF measurements such as age, gender, craniofacial morphology, periodontal support of teeth, signs and symptoms of temporo-mandibular disorders, tooth contacts, dental status and malocclusion (2-9).

Malocclusion can negatively affect the masticatory system (10). Magalhaes *et al.* (11) reported that malocclusion results in decreased masticatory performance, especially as it relates to a reduced occlusal contact area. Owens *et al.* (12) suggested that subjects with malocclusion have weaker bite force because they also have decreased areas of occlusal contact and near contact which decreases occlusal support.

Classification of occlusion in the permanent dentition describes the relationship of the maxillary and mandibular first molars while in the primary dentition, classification is based on the opposing primary second molar terminal plane relationship. Three types of occlusal pattern were reported in the primary dentition, flush terminal, mesial step and distal step (13).

Occlusal relationship in the primary dentition has been widely investigated. Racial predilection for certain molar relationships has been suggested (14). The flush terminal plane relationship was generally accepted as the norm for the completed primary dentition stage of occlusal development. Abu Alhaija and Qudeimat (15) reported that mesial step (48%) was the most common molar relationship in Jordanians followed by flush terminal relationship (37%). Anderson (14) concluded that in African American children as in European children, a mesial step, rather than a flush terminal plane, is the norm for the completed primary dentition.

As the occlusion of the permanent dentition is largely influenced by the framework provided by the preceding primary dentition, the conditions that may interfere with occlusal development should be considered. Up to our knowledge, the literature is deficient in studies on the relationship between occlusal patterns and OBF in the primary dentition stage, therefore, the aim of this study was to evaluate the OBF in pre-school children with different occlusal patterns.

## Material and Methods

Ethical approval for the study was obtained from the Institutional Review Board (IRB) at the XXXX University of Science and Technology (JUST). A randomly selected sample of 1085 kindergarten children in primary dentition stage were selected. The list that contained the names of schools enrolling the required age groups was obtained from the Directorate of Education in Irbid Governate. The age of subjects ranged from 3 to 6 years (averaged 4.90 ± 0.86 years). For each subject weight in kilograms and height in centimeters were recorded ( Table 1).

**Table 1:**
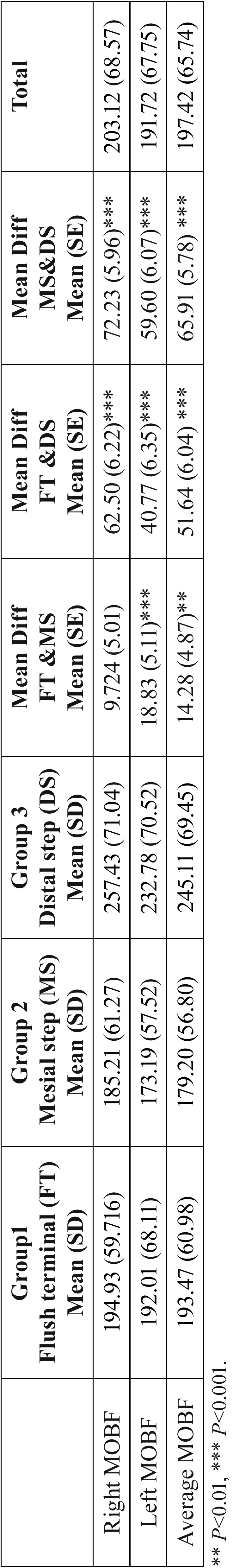
Means, standard deviations (SD), differences between means and standard errors (SE) of MOBF measurements in the three studied groups according to molar relationship.

Subjects included in this study were examined at their kindergartens. Using power analysis, it was calculated that at least 200 subjects in each group were required to detect a medium effect size (0.25 SD) between the five groups at a significance level of 0.05 with a power of 0.90.

The subjects were divided into three groups based on molar relation (13):

Group 1- Flush terminal molar relationship

The distal surfaces of the upper and lower second primary molars in the same vertical plane in centric occlusion. It consisted of 335 children (165 males and 170 females; average age 4.83 ± 0.88 years).

Group 2- Mesial step 

The distal surfaces of the lower primary second molar in anterior relationship to the distal surface of the upper second molars in centric occlusion. It consisted of 450 children (200 males and 250 females; average age 4.89 ± 0.85 years).

Group 3- Distal step 

The distal surfaces of the lower primary second molar in posterior relationship to the distal surface of the upper second molars in centric occlusion. It consisted of 300 children with (150 males and 150 females; average age 5.03 ± 0.83 years).

The inclusion criteria included the following:

1- Primary dention stage

2- No anterior or posterior crossbite or openbite.

3- No missing teeth in the regions of recording (molar area).

4- No local pain experienced at the deciduous molars.

5- No heavily restored teeth on the area of recording.

6- No gingival inflammation, no periodontal diseases and no mobility of the teeth.

7- No reported systemic disease (Chronic arthritis) or apparent facial asymmetry that could affect the recordings.

8- No soft tissue abnormalities.

9- No temporomandibular joint dysfunction.

Each child who fullfilled the inclusion criteria, received a consent form and an abstract of the study and its goals and method of examination. Those children who returned the consent form signed by their parents were examined.

The examination form consisted of 2 major parts: the demographic data part including the name of the child and the date of birth, ethnicity, height and weight and the measurement part including the measurement of OBF. Clinical examination involved the record of molar relationship, overjet, overbite and the presence of wearing facets. All the examinations were done by the same researcher (H.O.). Previous training for visual inspection of wearing facets was conducted in pediatric dentistry clinics.

The OBF was measured bilaterally in the second primary using a portable OBF gauge (GM10, Nagano Keiki, Tokyo, Japan). Subjects were seated upright without head support with the Frankfort plane nearly parallel to the floor. The OBF gauge consists of a hydraulic pressure gauge and a biting element made of a vinyl material encased in a polyethylene tube (16). Before the recording, subjects were trained to perform their highest possible OBF. Some behavioral difficulties were faced especially when taking the OBF measurements for children in groups 1 and 2. Children who did not manage to bite as instructed were excluded. OBF was measured alternately on the right and left sides with a 15 second resting time between each bite. Subjects were instructed to bite three times as hard as possible on the gauge without moving the head. The highest value of the three OBF measurements per side was recorded as the maximum occlusal bite force (MOBF) for that side. The mean value for the right and left sides was considered as the subject’s MOBF used in the analysis.

-The Null Hypothesis

OBF measurements in primary dentition are not affected by the different occlusal patterns.

-Method Error

All examinations were performed by the same examiner. To quantify the method error, 25 subjects (5 from each group) received the examination 2 times in 2 different occasions (one week interval). Kappa statistics was used to evaluate the errors in categorical data. Results of the Kappa values were above 80%, which indicates substantial agreement between readings. Dahlberg’s formula and Houston coefficient of reliability (17) were calculated for the MOBF measurement. Dahlberg error was 10 N and Houston coefficient of reliability was 84%.

-Statistical Analysis

Data analysis was carried out using the Statistical Package for Social Science (SPSS) computer software (SPSS 17.0, SPSS Inc., IL, USA). Descriptive statistics (means and standard deviation) for MOBF for each group were calculated. Gender differences were calculated using independent t-test. Analysis of variance (ANOVA) was used to determine whether significant differences existed between the studied groups. Bonferoni multiple comparison test was applied to identify which of the groups was different.

## Results

Means, SD, differences between means and standard errors (SE) of the MOBF measurements in respect to molar relationship, overjet and overbite are shown in  Table 2 and  Table 3. A statistically significant differences in the MOBF between the three groups were detected.

**Table 2:**
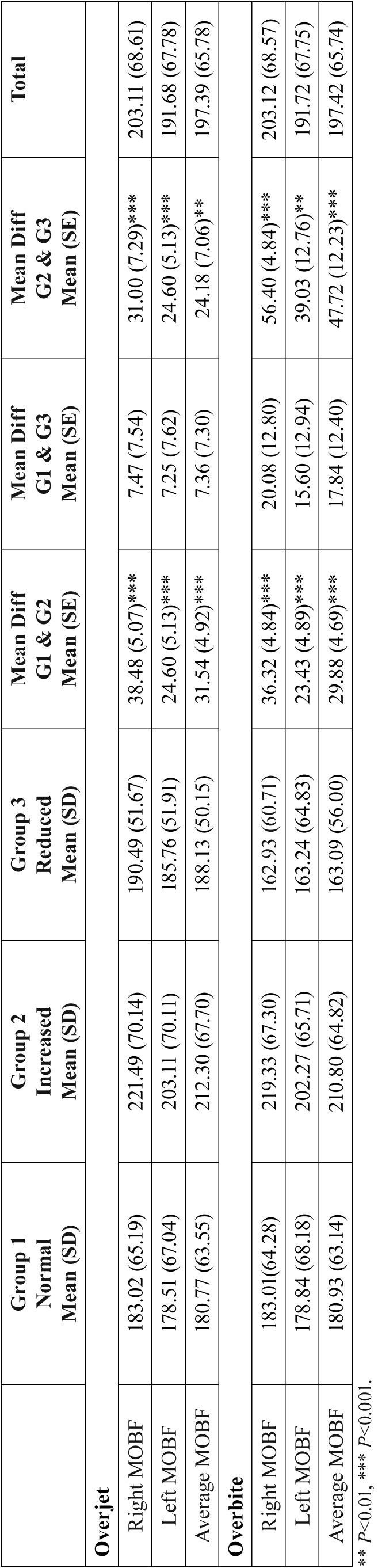
Means, standard deviations (SD), differences between means and standard errors (SE) of MOBF measurements in the three studied groups according to overjet and overbite.

**Table 3:**
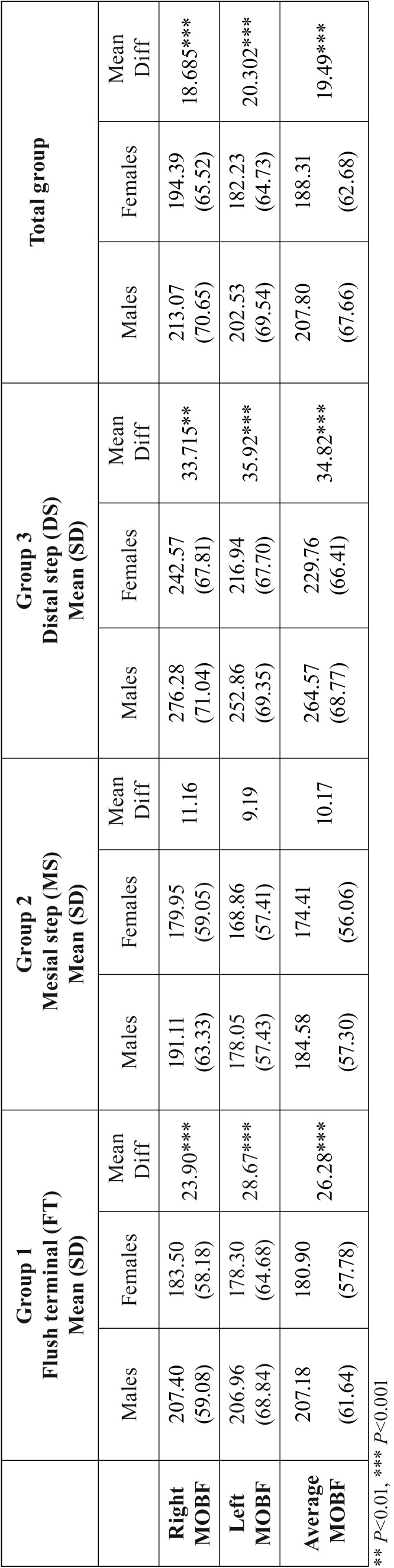
The means, standard deviations (SD), differences between means and significance in MOBF between males and females.

Gender differences were detected. Males had a stronger MOBF than females ( Table 4).

**Table 4:**
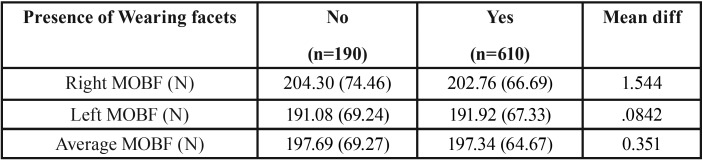
The means, standard deviations (SD), differences between means of MOBF (N) measurements between studies groups in respect to the presence of wearing facets.

Most of the subjects (76.5%) in this study had wearing facets on their teeth. Wearing facets were found in 212 subjects (63%), 400 subjects (89%) and 215 subjects (71%) who presented with flush terminal, mesial step and distal step primary second molar relationship. The difference between the three groups was statistically significant at *P*<0.001. No significant difference was detected in MOBF between subjects with and without wearing facets.

## Discussion

The mean MOBF in this study was 197 N which correspond closely with previously reported studies (9,18-20). Owais *et al.* (9) reported MOBF of 176 N in Jordanian pre-school children (169N in females and in 183N males).

In the current study, a correlation was found between gender and MOBF which was in agreement with previous studies (5,6,8,9,21). Males tend to have a higher MOBF than females in all groups. Ferrario *et al.* (3) explained the higher MOBF values in males by their larger dental size that present larger periodontal ligament areas and higher MOBF. Palinkas *et al.* (8) reported a 30% higher mean MOBF in males compared to females. In the contrary, other studies reported that gender is insignificantly related to OBF (23-25). Wichelhaus *et al.* (22) reported no significant differences in OBF between males and females. They suggested that it might be due to the small number of subjects included in their study and to the recording of functional forces that occurred during nocturnal sleep. Serra *et al.* (23) showed no gender effect on OBF in children from 6 to 9 years of age. Their study was performed on only 22 subjects using a pressurized tube transducer for bite force measurement. Ching-Ming *et al.* (24) reported that MOBF in males was larger than that of females, but that difference was not statistically significant. They explained that by the fact that the jawbone and masticating muscles of pre-school children are still in developing.

In this study, the MOBF was affected by the antero-posterior (A-P) occlusal relationship. MOBF was the highest in flush terminal and the lowest in mesial step molar relationships. This was in disagreement with Ching-Ming *et al.* (24) who reported that children with a flush terminal and mesial step molars had a higher OBF and MOBF on both sides than children with a distal step. Comparing the finding of the current study with previous studies in adult subjects revealed contradictory findings (4,25,26). Sonnesen and Bakke (4) compared the OBF in the different angle’s malocclusions. They found that the OBF was the lowest in Angle Class III malocclusion subjects (288.3 N) and the highest in Angle Class II malocclusion subjects (369.3 N). Throckmorton *et al.* (25) found that A-P relationships of the dentition were not correlated with MOBF. On the other hand, Roldán *et al.* (26) concluded that individuals with normal occlusion have a greater maximum bite force than do individuals with Class I or Class II malocclusion.

Ethnic differences and different OBF measurement techniques may explain the variations in the OBF measurements among the above mentioned studies.

Kamegai *et al.* (18) reported that decreased overjet is a significant factor affecting the OBF. In this study, MOBF was the highest in subjects with increased overjet (208.77 N). whereas, Throckmorton *et al.* (25) found that A-P relationships of the dentition were not correlated with MOBF. This finding is in parallel of the above finding of the current study that OBF was the highest in distal step molar relationship.

In this study, MOBF was the highest in subjects with increased overbite. These findings were consistent with those reported by van Spronsen *et al.* (27). On the other hand, Rentes *et al.* (28) stated that the type of occlusion in primary dentition do not affect the magnitude of OBF. However, the sample size was too small consisting of only 30 children and different device for measuring bite force was used. Also, Ching-Ming *et al.* (24) studied the relationship between the anteroposterior occlusal pattern and the vertical occlusal pattern with the MOBF in pre-school children. They reported that the OBF on both sides and the MOBF were the highest in open bite subjects and lowest in deep bite subjects. It should be noted that there were only four subjects in the open bite group, causing other factors to profoundly affect the final result.

The visual inspection of the dental wear is the most common tool of pediatric dentists to detect bruxism in children, but the reliability of this method has been questioned (29). In the current study, although 89% of subjects who presented with mesial step had wearing facets, these subjects had the lowest MOBF. It has been suggested that presence of wearing facets increase the surface area of occlusal contact. Larger occlusal contacts may be associated with fewer interferences, which permit a greater range of lateral excursion (30), thus, reducing the amount of OBF.

The findings of this study are important since they provide values for OBF in healthy young children with different occlusal patterns in the primary dentition. This data can therefore serve to provide reference values for use to understand the development of malocclusion in the succedaneous permanent dentition.

Limitation of this study includes different vertical skeletal relationships were studied within each A-P group.

## Conclusions

1. Children with distal step and mesial step molar relationship had the highest and the lowest MOBF, respectively

2. MOBF was the highest in subjects with increased over jet.

3. MOBF was highest in subject with deep bite.

4. MOBF was similar in children with/without wearing facets.
